# Quantitative Methods for Measuring Repair Rates and Innate-Immune Cell Responses in Wounded Mouse Skin

**DOI:** 10.3389/fimmu.2018.00347

**Published:** 2018-02-27

**Authors:** Zhi Li, Elizabeth Gothard, Mark C. Coles, Carrie A. Ambler

**Affiliations:** ^1^Biosciences Department, Biophysical Sciences Institute, Durham University, Durham, United Kingdom; ^2^Centre for Immunology & Infection, Department of Biology and Hull York Medical School, University of York, York, United Kingdom; ^3^Kennedy Institute of Rheumatology, University of Oxford, Oxford, United Kingdom

**Keywords:** flow-cytometry methods, innate immunity, skin, wound, 3D imaging

## Abstract

In skin wounds, innate-immune cells clear up tissue debris and microbial contamination, and also secrete cytokines and other growth factors that impact repair process such as re-epithelialization and wound closure. After injury, there is a rapid influx and efflux of immune cells at wound sites, yet the function of each innate cell population in skin repair is still under investigation. Flow cytometry is a valuable research tool for detecting and quantifying immune cells; however, in mouse back skin, the difficulty in extracting immune cells from small area of skin due to tissue complexity has made cytometric analysis an underutilized tool. In this paper, we provide detailed methods on the digestion of lesion-specific skin without disrupting antigen expression followed by multiplex cell staining that allows for identification of seven innate-immune populations, including rare subsets such as group-3 innate lymphoid cells (ILC3s), by flow-cytometry analysis. Furthermore, when studying the functions of immune cells to tissue repair an important metric to monitor is size of the wound opening. Normal wounds close steadily albeit at non-linear rates, while slow or stalled wound closure can indicate an underlying problem with the repair process. Calliper measurements are difficult and time-consuming to obtain and can require repeated sedation of experimental animals. We provide advanced methods for measuring of wound openness; digital 3D image capture and semi-automated image processing that allows for unbiased, reliable measurements that can be taken repeatedly over time.

## Introduction

Skin is an essential protective barrier that covers the surface of our body (1). It is composed of two main compartments, the surface epithelium formed of tightly aligned, stratified keratinocytes that give rise to the water proof surface barrier preventing microbial entry and the second compartment, the dermal compartment comprised of fibroblasts, peripheral nerves, blood vessels, muscle, and fat that provide strength, sensory function, and oxygenation. Both the epidermis and dermis contain numerous skin-resident immune cells including innate lymphoid cells (ILC3s), adaptive and regulatory T cells, and specialized antigen-presenting cells (APCs), e.g., Langerhans cells, dendritic cells (DCs), monocytes, and macrophages (2–5). These specialized skin-resident and infiltrating leukocytes have long been implicated in contributing to the regulation of all normal skin functions including steady-state homeostasis and normal tissue repair (3, 6–8). Furthermore, in skin pathologies (e.g., psoriasis, lupus erythema, and carcinomas) immune cell functions go beyond being complicit but have clear roles in both the initiation and progression of these pathologies (2, 6, 7, 9–14).

Epithelial and skin immune cells communicate through release of cytokines and growth factors and normal tissue repair requires elegant coordinated communication between these cell populations to enable healing to occur in a staged process (15, 16). Following injury, phagocytic cells infiltrate the skin to clear microbial contaminants. Following this initial phase, granulation tissue forms, dermal fibroblasts become contractile and produce new extracellular matrix proteins, and the epidermal keratinocytes proliferate and migrate to close the break in the epithelial barrier. In the final phase, the newly closed wound site undergoes tissue remodeling to help restore, albeit not fully, the strength and stability of the injured tissue. At all wound stages, adaptive and innate-immune cells work in concert with the epithelial cells through production of cytokines, anti-microbial peptides, and other growth factors.

Flow cytometry is a widely used technique to examine immune cells in many tissues in the body. However in skin, flow-cytometric analysis of immune populations has had limited uptake. Reasons include poor recovery of immune cells from skin tissues, lengthy digestion requirements for isolation of tightly connected epithelium, and extracellular matrix-rich dermal compartment, and in the case of wounds, limitations in size and number of available skin biopsies making analysis of local immunity even more challenging. One skin area where cell isolation can be performed efficiently is the ear (17), where hair follicles are shallower and the dermis is thinner. However, ear skin is not suitable for studying full thickness wound healing repair due to its distinct anatomy; ear cartilage is sandwiched by double-sided skin. Existing, published protocols for isolation of immune cells from full thickness, back skin mouse wounds require multiple enzymatic steps. These include an initial overnight Dispase I digestion followed by additional treatment with collagenase/hyaluronidase exposure to achieve immune cell isolation (18). However, there is scope for method advancement; it is known that the duration of enzyme exposure impacts cell surface protein expression and viability and that exposure to high concentrations of Dispase I causes significant loss of cell surface protein expression (19).

Recently, published work from several groups have provided new rapid, one-step methods for cell isolation, and flow-cytometric analysis of immune cells from more complex tissues including normal back skin and immune challenged skin (17, 20). Here, we add to this newly emerging repertoire and offer detailed methods for flow-cytometric analysis of leukocyte populations in small-tissue biopsies of highly inflamed back skin wounds. We provide methods for rapid cell isolation using minimal preprocessing of isolated skin tissue and a previously unreported enzyme cocktail Liberase^TM^/Collagenase D.

Furthermore, we provide new, standardized procedures for wound creation and monitoring closure using non-invasive 3D imaging. Current practices for wound measurement using calipers are subjected to human bias and require significant and repeated interventions (e.g., anesthetization) to experimental animals. Camera-based techniques have been shown to provide accurate measurements of human punch-biopsy wounds, burns, and diabetic ulcers single-time points (21–24). An additional study, where the same wounds were repeatedly imaged at irregular intervals, suggested that non-invasive imaging could provide accurate measurements of wound closure (25). Here, we detail image capture and software analysis using a 3D camera system to provide quantitative imaging with a high level of inter-operator agreement.

## Materials and Equipment

**Figure 1:**
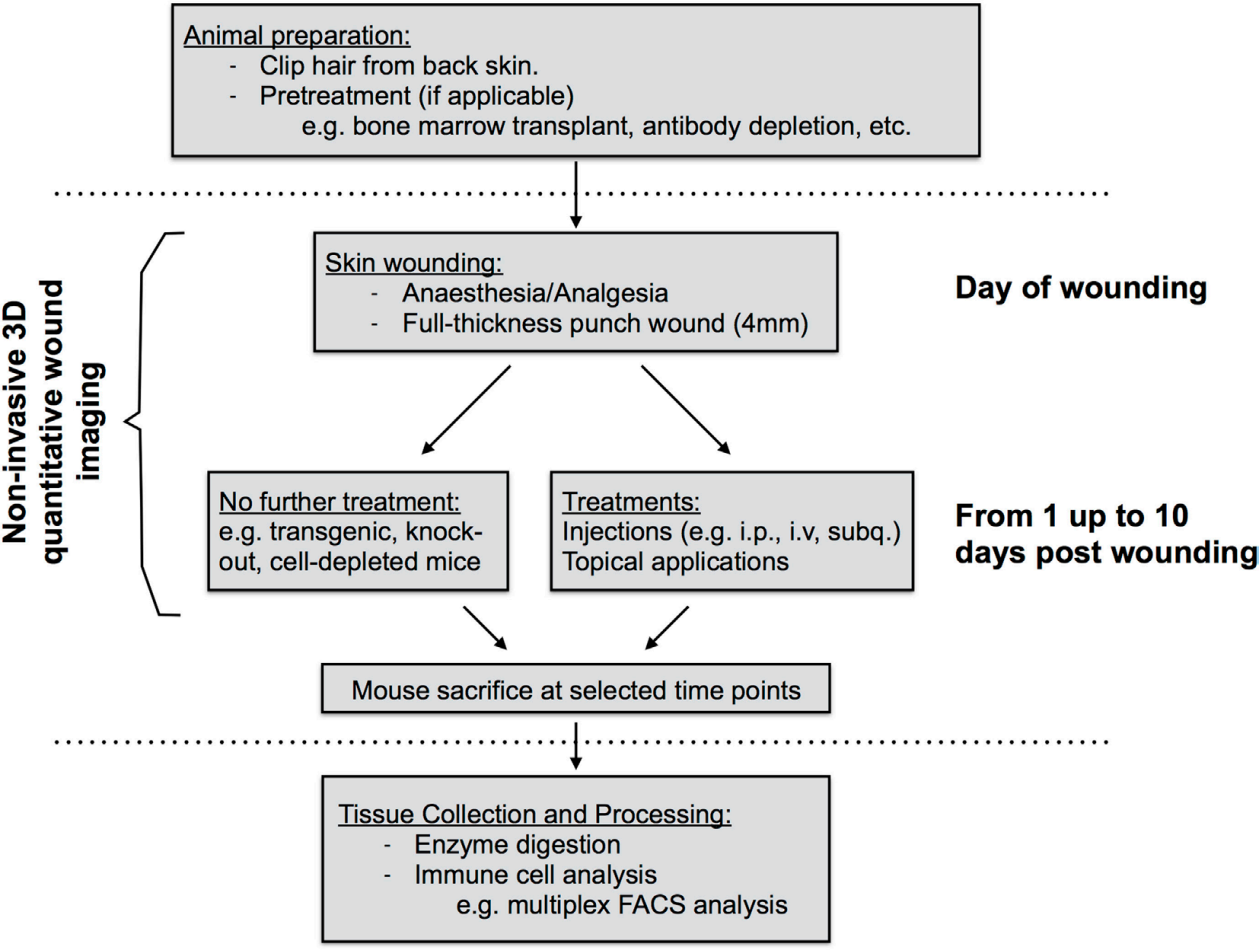
Diagram of work flow of mouse skin wound experiment procedures. Prior to wounding, hair must be clipped from the back skin. Common pretreatments can also include whole-body irradiation followed by bone-marrow transplantation or antibody depletion of cell populations prior to wounding. On the day of wounding and all subsequent days, wounds are imaged non-invasively using 3D image capture and may be exposed to further treatments if required. At sacrifice, back skin wound tissues are collected, subjected to enzyme digestion, and isolated cells processed for analysis by flow cytometry.

This study was carried out in accordance with the recommendations of the Life Sciences Ethical Review Process (Durham University) and Animal Welfare and Ethical Review Body (University of York) panels in accordance with guidance from the Home Office (UK). The protocol was approved by the Home Office UK in project licenses awarded to 60/3941 (Ambler) and 60/4178 (Coles). Wild-type C57/bl6 were used. Some mice received a pretreatment (e.g., bone-marrow transplant, antibody depletion, etc.) prior to wounding. Pretreatment methods have been used previously in the published literature (Figure 1). Antibodies were obtained from the commercial suppliers listed with in Table 1.

**Table 1:** List of antibodies, suppliers, and dilutions.

Antibody	Clone	Color	Dilution	Supplier	Cat no.
CD3	145-2C11	BV605	1:100	BD	563004
CD3	17A2	PE-Cy7	1:100	Biolegend	100220
CD4	RM4-5	FITC	1:100	eBioscience	11-0042-82
CD11b	M1/70	PE-Cy7	1:200	Biolegend	101216
CD11c	HL3	BV421	1:100	BD	562782
CD25	PC61.5	PE	1:100	Biolegend	102007
CD25	PC61.5	PE-Cy7	1:100	eBioscience	25-0251-82
CD45	30-F11	PerCP-Cy5.5	1:100	Biolegend	6264439
CD90.2	53-2.1	PE	1:100	Biolegend	140307
CD127	SB/199	PE-CF594	1:100	BD	SB/199
CD127	A7R34	efluor 660	1:100	eBioscience	51-1271-80
CCR6	29-2L17	PE	1:100	Biolegend	129803
CXCR4	L276F12	PE	1:100	Biolegend	146505
F4/80	BM8	efluor 660	1:100	eBioscience	50-4801-82
Lineage	17A2, RA3-6B2, M1/70, TER-119, RB6-8C5	efluor 450	1:100	eBioscience	88-7772-72
Ly6C	AL-21	PE-CF 594	1:200	BD	562728
Ly6G	1A8	FITC	1:100	Biolegend	127606
MHCII	M5/114	V500	1:200	BD	562366
NK1.1	PK136	PE	1:100	Biolegend	108707
RORγt	Q31-378	Alexa fluor 647	1:100	BD	562682
ST2	RMST2-2	APC	1:100	eBioscience	17-9335-80
Viability dye		efluor 780	1:1,500	eBioscience	65-0865-14

## Stepwise Procedures

### Creation of Surgical Wounds

**Figure 2:**
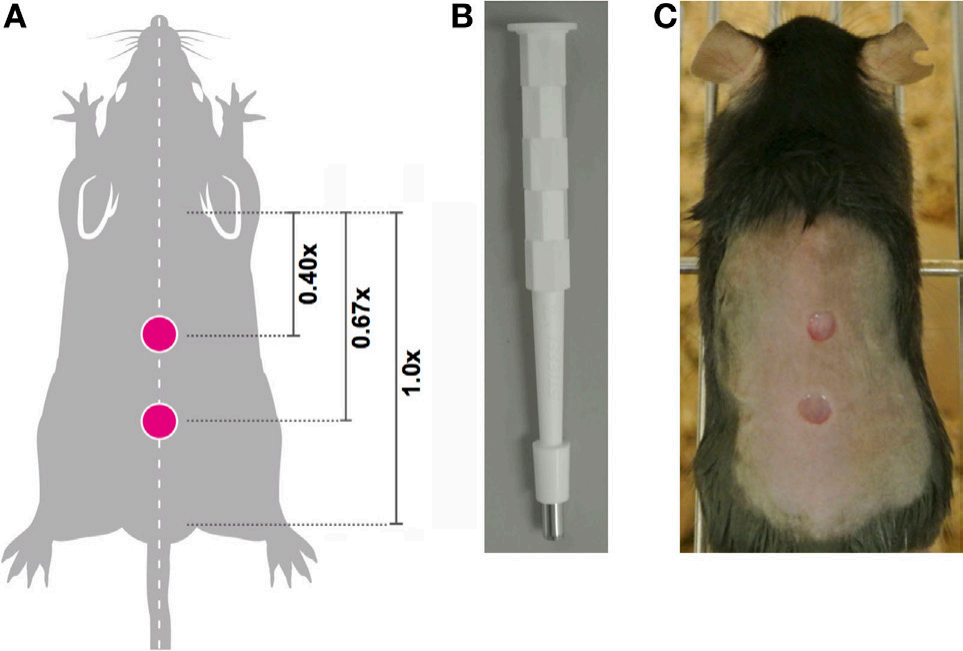
Location of midline wounds. **(A)** The distance between the base of the ears equals a 1x distance. To ensure consistency in wound creation, two 4-mm, full thickness wounds were created at 0.4× and 0.67× the ear-to-tail distance in each animal. **(B)** 4-mm surgical punch biopsies were used to create full thickness wounds. **(C)** An anesthetized mouse with two midline wounds; image captured immediately post-wounding.

Prior to wounding, each animal was assigned a unique identifier. One day prior to wounding, the hair on the dorsum was removed using clippers being careful not to nick or injure the skin. Each mouse was given systemic analgesic by subcutaneous injection (Vetgesic; 0.05 mg/kg) 30 min prior to surgery. Mice were anesthetized using inhaled isofluorane and the dorsal back skin was sterilized by topical application of Povidone-iodine solution 10% w/w (Videne), followed by washing three times with sterile water. Next, the distance between base of ears and base of tail was measured and dual, full thickness wounds were created along the dorsal midline at 0.4× and 0.67× the ear-to-tail distance using a 4-mm surgical punch biopsy (Stiefel) (Figure 2). Following surgery, mice were monitored during recovery and then daily to monitor wound closure.

### Quantitation of Wound Closure Using 3D LifeViz Micro™ Camera

**Figure 3:**
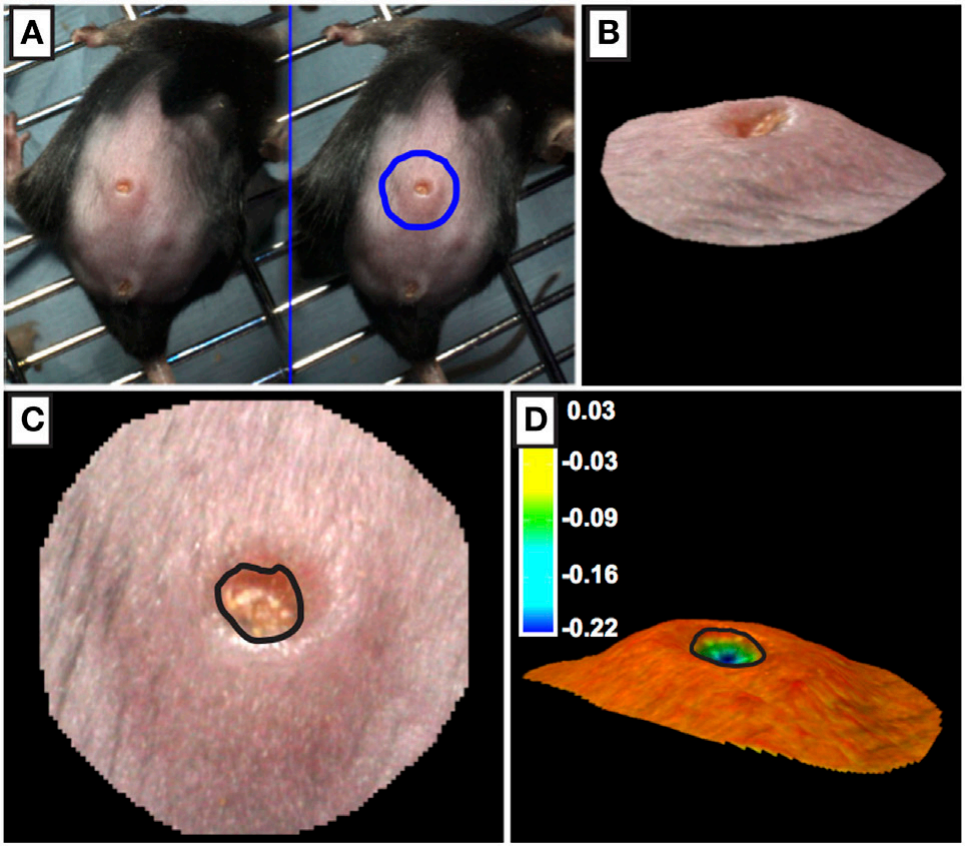
Processing of 3D images of wounds. **(A,B)** The outer periphery of the wound site was outlined using the circle function with the circle diameter was approximately twice the diameter of the wound. **(C,D)** The edge of the wound (marked by black line) was calculated using topographical criteria using QuantiCare Dermapix Pro software and is shown in normal view **(C)** and pseudo-colored contour view **(D)** Pseudo-colored scale in microns.

Three photographs of the wound site were captured daily using a 3D LifeViz Micro™ camera. Image capture required two staff: one to restrain the animal and the second to operate the camera. The mouse was restrained with gentle pressure applied holding the tail away from the head to present the wound site. Care was taken to not stretch the skin beyond its normal tension. Blinded analysis was carried out; each photograph was assigned an anonymized, random identifier prior to analysis. Photographs were imported into QuantiCare Dermapix Pro software and opened using *Visualization* tab (Figure 3). The circle function was used to outline the wound site outer periphery; the circle diameter was approximately twice that of the wound margins. Next, the *3D mesh reconstruction* was run followed by processing in the *3D analysis mode*. In the *3D analysis mode* with the *Render Options 2D* turned off, the contours were pseudo-colored allowing for visualization of wound margin. To quantify wound “openness,” results were calculated in the following way: under the *Meshes* tab, *Close volume* and *wound* were ticked and that *sigma value was* set to 1. *Difference* was selected in the drop-down menu under *Result* and a table of wound metrics was copied and saved to an excel spreadsheet. Finally, results were unblinded and statistical analysis carried out using *R*.

#### Isolation of Immune Cells for Flow-Cytometry Analysis

Following humane euthanasia, the whole back skin was removed and placed onto a nitrocellulose membrane. Using a scalpel, the skin was cut transversely between the two wounds, subsequent cuts were made resulting in a square of tissue of approximately 8 mm × 8 mm that included the wound and adjacent tissue. Next, the tissue was removed from the membrane and subcutaneous fat and muscle was cut from the dermal surface. Tissue was briefly rinsed in 1× PBS and was cut the tissue into 4–5 smaller pieces before placing the pieces in a 1.5-mL Eppendorf tube containing a Liberase TL enzyme cocktail [0.35-mg/mL Liberase TL (Roche, cat. #5401020001), 3-mg/mL Collagenase D (Roche, cat. #11088866001), and 0.1-mg/mL DNase I (Roche, cat. #10104159001)], or Dispase II enzyme cocktail (2 mg/mL, Thermo Fisher 17105-041 and 0.1-mg/mL DNase I) in a volume of 700-µL RPMI-1640 (Thermo Fisher, 31870-025) media and incubate on a block heating shaker at 1,400 rpm, 37°C for 2 h. After incubation, remove undigested debris by filtering the sample through the side of a 70-µm strainer into a new collection Eppendorf tube. Add 500 µL of cold FACS buffer (0.5% BSA; 2-mM EDTA in PBS) to the side of strainer to wash off the remaining cells into the collection tube. Centrifuge at 4°C for 5 minu at 400× *g*. Remove the supernatant and resuspend the cell pellet in 200-µL cold FACS buffer. Count cells and transfer 0.5–1 × 10^6^ cells into the assigned well in a 96-well V-bottom plate. Keep the plate on ice and proceed to one of the staining procedures described below. Pool cells from multiple wounds as required.

#### Extracellular and Intracellular Antibody Staining for Flow Cytometry

Make a master mix for blocking solution: anti-CD16/CD32 and rat IgG diluted at 1:100 and 1:1,000, respectively, in 50-µL cold FACS buffer per sample. After centrifuging, resuspend the cells in each well with 50-µL blocking solution to block non-specific binding and incubate for 20 min at 4°C. Following incubation, centrifuge the plate at 300× *g* at 4°C for 5 min and discard supernatant. Rinse 1× with FACS buffer and add diluted antibodies [50 µL per well; anti-mouse CD45 1:100, lineage cocktail 1:100 (consisting of CD3, B220, TER-119, CD11b, Gr-1), CD127 1:100, fixable viability dye 1:1,500]. Incubate for 30 min at 4°C. Centrifuge as above and wash cells in 200-µL cold FACS buffer per well three times pelleting cells by centrifugation in each wash step prior to discarding supernatant. Upon completion of extracellular antibody staining, cells were either analyzed immediately or additionally processed to detect intracellular antigens as described below.

For intracellular staining, resuspend pelleted cells in 100-µL 1× FACS fixation buffer (eBioscience, 00-5521; made according to manufacturer’s instructions) incubated overnight at 4°C. Pellet cells by centrifugation and wash cells in cold FACS buffer per well as described above. To detect RORγ, resuspend pelleted cells in 50-µL RORγt antibody diluted in intracellular staining buffer [1× permeabilization buffer (eBioscience, 00-8333) diluted in FACS buffer (see above)]. Incubate for 1 h at 4°C, the centrifuge plate and discard supernatant. Wash in cold FACS buffer as above then resuspend cells in 400-µL cold FACS buffer, transfer into pre-labeled FACS tubes and analysis.

## Results

### Quantifying Wound Healing

We developed a robust, reproducible, and quantitative wound-healing protocol that would allow us to compare result groups that had been treated in different ways including analyzing normal mice pretreated by targeting immune cells through irradiation or antibody-depletion (workflow summarized in Figure 1). Factors impacting closure rates include wound location and the stage of the hair growth cycle (26, 27). Therefore in all cases, young mice between 7 and 10 weeks of age were used and 4-mm punch wounds were introduced using a sterile punch biopsy at sites 0.4× and 0.67× the distance between the posterior edge of the ear and the start of the tail (distance designated as 1×; Figures 2A–C). Prior to wounding, back skin hair was clipped near the base of the skin with clippers taking care not to injure the skin. Following surgical wounding, wound sites were left uncovered for the remainder of the experiment allowing for topical application of chemical activators/inhibitors directly to the wound site at defined time points post wounding.

To quantify wound-closure photographs of wounds were taken daily using a 3D LifeViz™ camera. A focused image was captured at a standardized distance by using the camera’s dual-beam red light pointers on the wound site, positioning the camera such that the beams were entirely overlapping and circular (as opposed to elliptic) ensured the picture was taken from the correct distance and angle. We found that images with excessive reflective glare in the wound area gave inaccurate results after analysis and therefore were discarded. Following image capture, blinded images were uploaded into the software and scored. We found that using the wound topographical characteristics we could accurately measure quantify openness of the wound. The semi-automated software analysis ensured that measurements were reproducibly calculated by several users [Figure 3; Ref. (8)]. Previously studies needed to quantify wound openness following in live, anesthetized animals or following euthanasia. However, in our model we can take repeated wound measures by using non-invasive 3D camera when animals are conscience and under minimum stress.

### Immune Cell Isolation and Flow Cytometry

**Figure 4:**
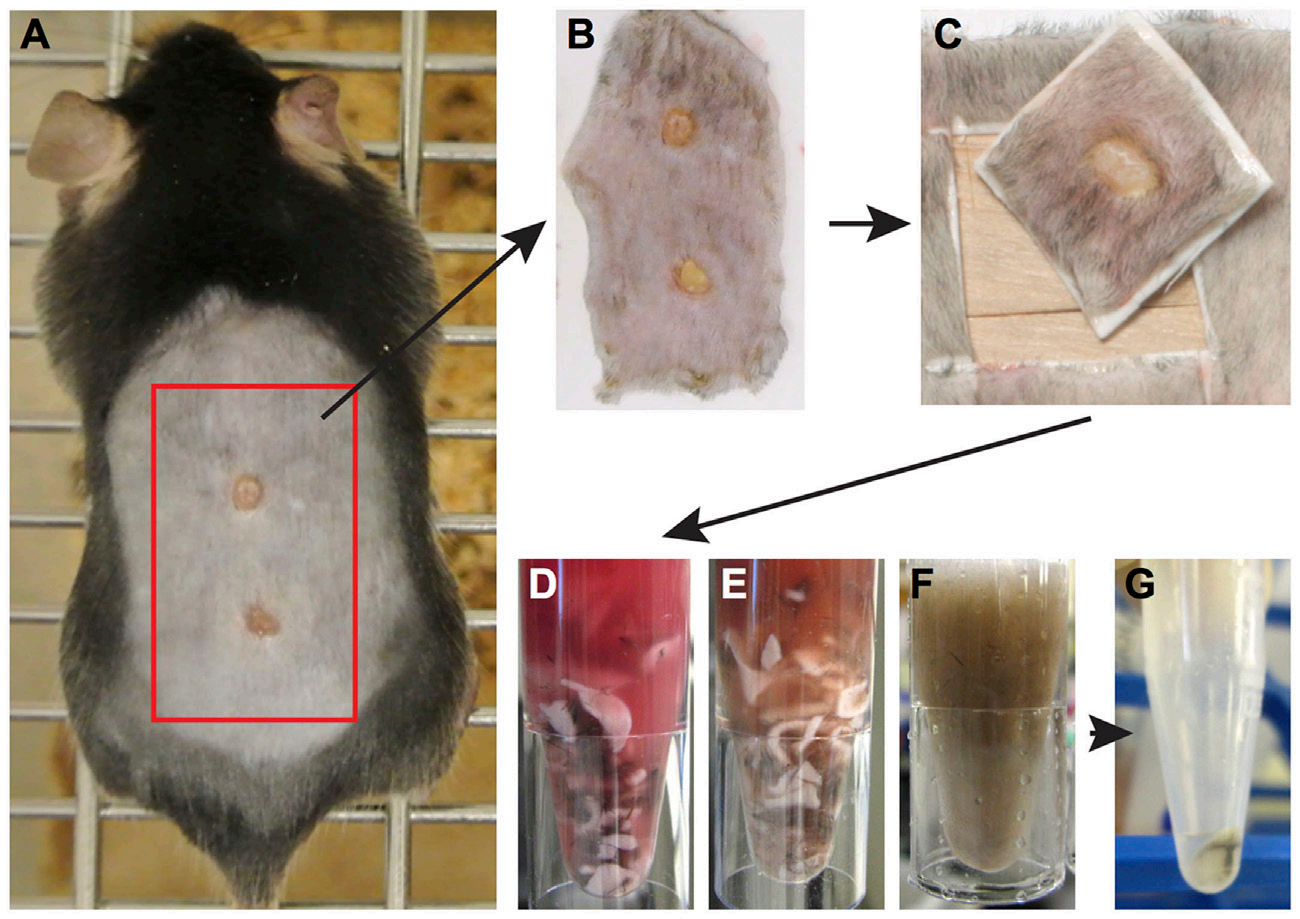
Enzymatic tissue digestion. **(A)** Whole-body photograph of wounded mouse just prior to sacrifice. **(B)** Skin tissue removed from sacrificed animal and placed on white filter paper to prevent folding of tissue. **(C)** 1 cm^2^ piece of skin containing the wound and surrounding tissue. Note wound should be centered within the isolated tissue. **(D–F)** Tissue cut into small pieces with a scalpel placed in a 1.5-mL Eppendorf tube containing an enzyme digestion cocktail. Tissue digestion takes place at 37° for 2 h and images show tissue digestion at the start **(D)**, midway through digestion, **(E)** and at the completion of digestion **(F)**. **(G)** Following digestion, the solution is filtered to remove tissue debris and remaining cells are pelleted by centrifugation. Cells are now ready for analysis by flow cytometry.

**Figure 5:**
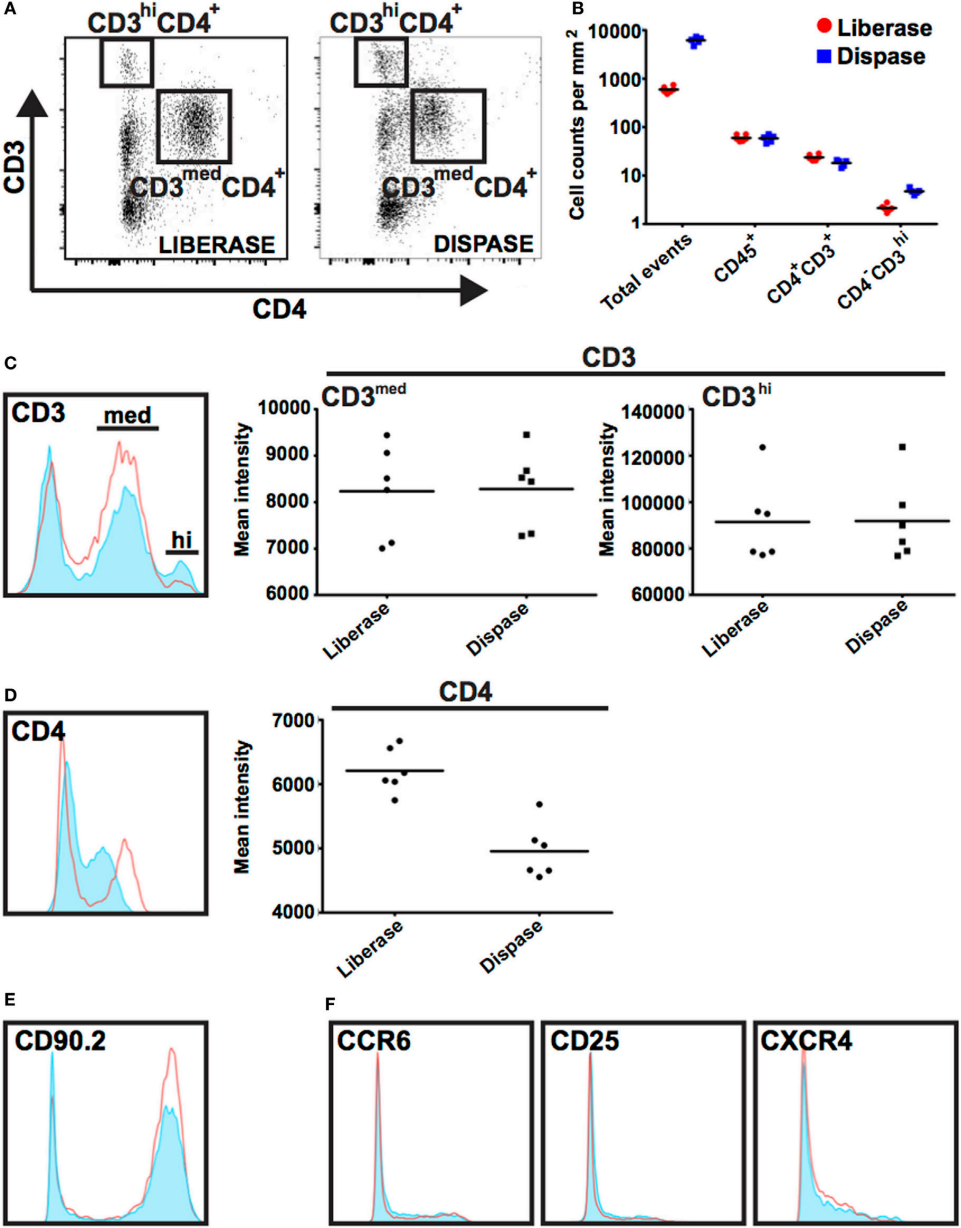
Impact of Dispase and Liberase/Collagenase D digestion methods on populations of immune cells. **(A)** Single-cell suspensions were prepared using Liberase TL/Collagenase D (Liberase) or Dispase enzyme cocktails and isolated cells co-stained with antibodies prior to analysis by flow cytometry. Cells were initially gated based on CD45 expression and subsequently gated on CD3 and CD4 expression as shown. CD3^hi^ CD4^−^ expression marks dendritic epidermal T cells (DETCs) located in mouse epidermis and CD3^+^ CD4^+^ expression is detected on dermal infiltrating T cells. **(B)** Graph representing the number of events as counted by flow cytometry in a 1 mm^2^ area of wound tissue. Note tissue digestion using Dispase leads to an approximate 10-fold increase in total cell numbers; however, the number of CD45^+^ immune cells was similar regardless of digestion method. **(C–F)** Histograms show expression of cell surface markers in gated Dispase II digestion (red line) and Liberase TL/Collagenase D digestion (filled light blue) populations. With the exception of CD4, there was no detectable difference in intensity of surface marker staining.

Inflammation plays key roles in all phases of wound repair, but early inflammation response is largely driven by influx of innate-immune cells. To analyze immune cells in skin wounds, a 1 cm^2^ area including the wound and surrounding skin was excised from the back skin. Immune cells were isolated by digestion from skin wounds. It was important to analyze a consistent area of skin to allow experimental results to be pooled and compared between treatments or genotypes. We tested whether Liberase TL/Collagenase D cocktail or Grade II Dispase (Dispase II) allowed for full and complete digestion of the dermal tissue (Figures 4A–G). However, we detected significant differences in the resulting cell populations. Dispase II digestion yielded 10-fold higher cell counts overall compared with Liberase TL/Collagenase D digestion with a 20-fold greater number of CD3^hi^CD4^−^ dendritic epidermal T cells (DETCs), suggesting that Dispase II may act more effectively in isolation of epidermal keratinocytes and immune cells. Dispase digestion is known to be effective for liberating epidermal cells (28); thus, epidermal epithelial cells make up the bulk of the additional cell numbers in the Dispase II digestion sample. Consistent with this, the number of epidermal-resident DETCs (CD3^hi^ CD4^−^) was also higher than in the Liberase TL/Collagenase D digestion sample further suggesting that Dispase II is more effective at breaking down the skin epidermal layers. Interestingly, the total number of CD45^+^ immune cells was the same in both methods (Figures 5A,B) and strikingly, the expression level of CD4, but not other surface proteins, was reduced significantly by Dispase II digestion (Figures 5C–F). Consistent with this finding, it has been previously reported that Dispase I digestion of splenocytes reduced expression of CD4 antigens (19).

**Figure 6:**
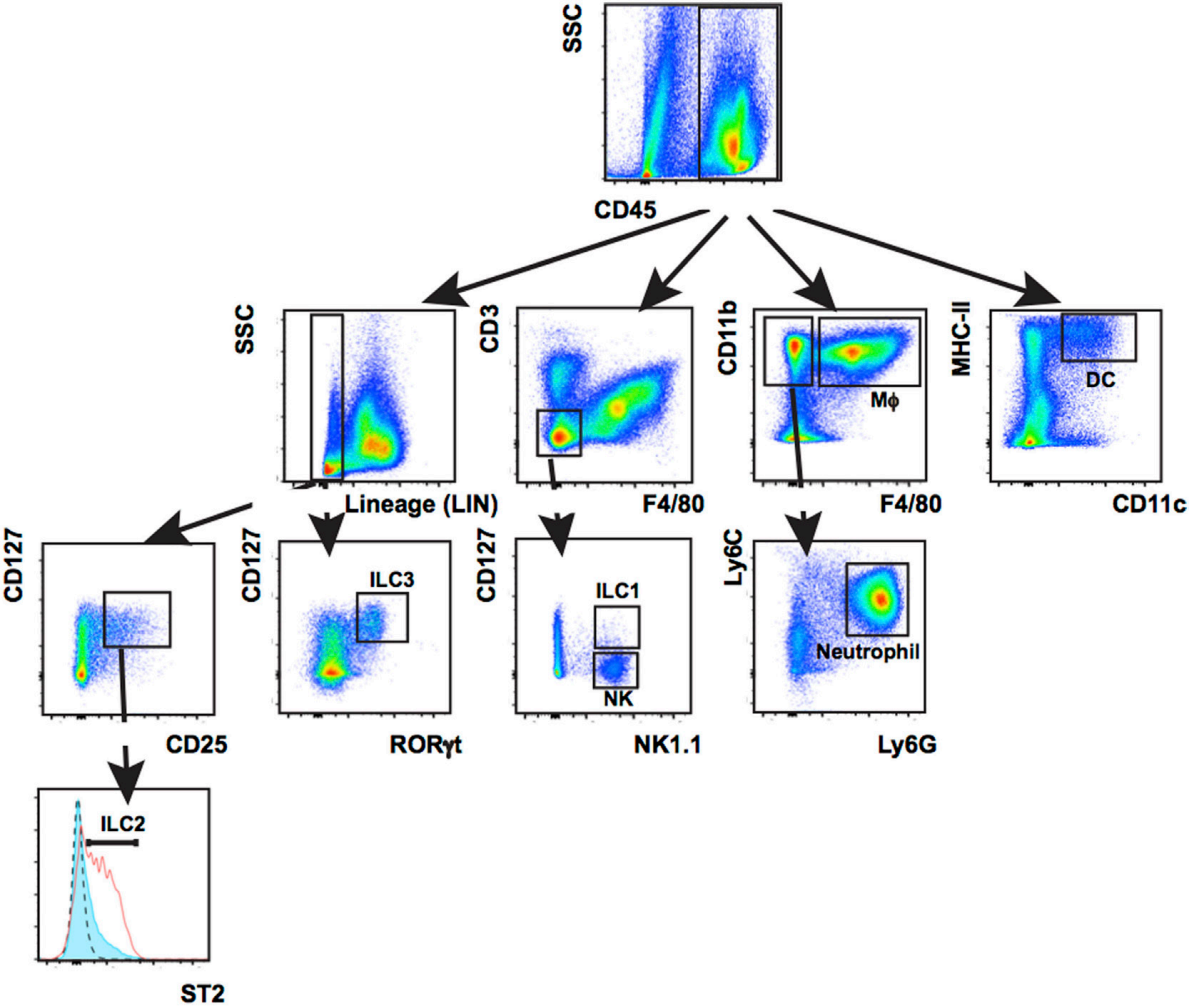
Leukocyte populations present in wounded back skin quantified by flow cytometry. Back skin wounds collected 5 days post wounding were digested with Liberase TL/Collagenase D cocktail to a single-cell suspension and the cells were labeled with antibodies to CD45, Lineage cocktail of antibodies (Lin) CD3, CD11b, CD11c, MHC-II, Ly6C, Ly6G, F4/80, NK1.1, CD127, ST2, and RORγt prior to analysis. Labeled cells were gated first on CD45 and then subsequently as shown. This multiplex gating strategy allows for efficient identification of seven innate-immune cell subtypes (Mϕ, macrophage; DCs, dendritic cells; NK, natural killer cells; ILCs, group 1, 2, and 3 innate lymphoid cells; neutrophil) from the same sample. This technique is highly suitable when needing to analyze immune populations in a localized, small sample such as a wound.

Innate-immune cells including monocytes/macrophages, neutrophils, and ILC3s are essential responders to tissue damage to help clean up infection and facilitate healing. We characterized innate-immune cell responses at wound sites using multiplex phenotypic characterization by flow cytometry to quantify cell subsets. Multiplex analysis cells was carried out with the surface markers CD3, CD11b, CD11c, MHC-II, F4/80, Ly6c, Ly6G, CD127, NK1.1, ST2, or CD25 together with CD45 and the viability dye efluor780 (eBiosciences) which is compatible with cell fixation. After cell surface labeling, cells were fixed, permeablized, and stained with antibodies to the transcription factor RORγt specific for ILC3s and T-cell subsets before flow analysis. Among CD45^+^ cells, DCs were detected by CD11c^+^ MHC-II^+^, macrophages by CD11b^+^ F4/80^+^, neutrophils by CD11b^+^ F4/80-Ly6G^+^, NK by CD3-F4/80-CD127-NK1.1^+^, ILC1 by Lin-CD3-F4/80-CD127^+^ NK1.1^+^, ILC2 Lin-CD127^+^ CD25^+^ ST2^+^, and ILC3 Lin-CD127^+^ RORγt^+^. Percentage of individual subpopulations as R1*R2*R3 were calculated (R1 = percentage of CD45^+^ cells in total cells, R2 = percentage of gated cells in CD45^+^ cells, and R3 = percentage of gated cells in R2; Figure 6).

## Concluding Remarks

We present new methods for quantifying wound closure in 3D in live animals and for multiplex analysis of leukocyte populations from wound dermis and epidermis using flow cytometry. These techniques offer several advantages over existing techniques. 3D imaging is non-invasive and limits the need for repeated animal sedation. The data are highly reproducible and are processed blinded, thus limiting potential experimental bias. Like this study, 3D camera imaging has been used for objective assessment of the rate of healing in burns patients (29). Furthermore, digital measurements are calculated from captured images providing robust and reproducible methods to collect, analyze, and store scientific data; these practices align with good data management guidance outlined by major government research institutions (30, 31). Our laboratories have validated the effectiveness of these non-invasive methods by examining wound-healing defects in innate-immune cell depleted mouse strains (RORγ^−/−^) compared with controls. Image analysis revealed that RORγ^−/−^; mutant mice heal slower and parallel immune cell phenotyping in cells isolated and analyzed as described above showed delayed immune cell responses in these animals. Using these analyses, we were able to correlate the state of wound closure (by calculating wound openness using perimeter measurements) with the relative populations of leukocytes in the wounds at different time points in the healing cycle (8).

Leukocyte analysis by flow cytometry is the gold standard. However, in skin wounds the use of flow cytometry has been hampered by the difficulty in isolating leukocytes with intact cell surface receptors from the extracellular matrix-rich wound stroma and granulation tissues and researchers have often relied upon more subjective methods including antibody localization on sectioned tissues. Additional problems arise from the digestion enzymes themselves as some enzymes, e.g., Dispase I, cleave cell surface proteins having direct consequences for phenotypic analysis (19). Building upon this work, new Dispase-free cell isolation methods have been developed for cell isolation of less-complex tissues including normal and inflamed skin and other organs (17, 20). Here, we have demonstrated effective cell isolation methods using a Liberase TL/Collagenase D cocktail compatible with standard immunological assays based on cell surface marker expression providing a direct readout of immune cell populations in from epidermal and dermal wounds tissues.

In this study, we also tested the impact of wound digestion with Dispase II and found that similar to Liberase TL/Collagenase D enzymes, Dispase II enzymes were efficient at digesting the tissue fully. Both treatments liberated similar numbers CD45+ leukocytes from wounds; however, Dispase digestion was superior in the release of CD3^hi^ CD4^−^ from the epithelial layers. Dispase II digestion also had greater impact cell surface protein expression; however, we only found significant reduction of detectable CD4 antigen. In summary, both enzymes allowed for isolation of wound-resident immune cells compatible with standard flow-cytometry analysis.

## Ethics Statement

This study was carried out in accordance with the recommendations of the Life Sciences Ethical Review Process (Durham University) and Animal Welfare and Ethical Review Body (University of York) panels in accordance with guidance from the Home Office (UK). The protocol was approved by the Home Office UK in project licenses awarded to 60/3941 (Ambler) and 60/4178 (Coles).

## Authors Contributions

ZL, EG, CA, and MC designed experiments. ZL, EG, MC, and CA performed experiments and analyzed data. ZL, EG, and CA produced the figures. ZL, EG, MC, and CA wrote the manuscript.

## Conflict of Interest Statement

The authors declare that the research was conducted in the absence of any commercial or financial relationships that could be construed as a potential conflict of interest.
